# Prevalence of Dysphagia and Its Health Implications Among Elderly Residents in Long-Term Care Facilities in the Liguria Region (Italy): An Observational Cohort Study

**DOI:** 10.3390/nu17203268

**Published:** 2025-10-17

**Authors:** Elena Formisano, Enrico Di Cino, Elena Nicosia, Andrea Pasta, Gianfranco Paccione, Alessandro Antioco Sukkar, Livia Pisciotta, Samir Giuseppe Sukkar

**Affiliations:** 1 Department of Internal Medicine, University of Genoa, 16132 Genoa, Italy; 2 Dietetics and Clinical Nutrition Unit, IRCCS Policlinic Hospital San Martino, 16132 Genoa, Italy; 3 Department of Health and Social Services, Liguria Region Administration, Piazza della Vittoria 119, 16121 Genova, Italy; 4 Farmacy Department, Istituto Don Orione di Genova, 16143 Genova, Italy; 5 Facoltà di Medicina e Chirurgia, Università Cattolica del Sacro Cuore, 00168 Rome, Italy

**Keywords:** dysphagia, malnutrition, body composition, sarcopenia, elderly population

## Abstract

**Background/Objectives**: Dysphagia is a common condition among older adults, associated with significant health risks. This prospective, open-label observational cohort study aimed to determine the prevalence of dysphagia and its impact on nutritional status and clinical outcomes in elderly residents of six long-term care facilities. **Methods**: Patients aged ≥ 65 years were screened using the 3 oz Water Swallow Test (WST); those with dysphagia were followed for 6 months. Nutritional status was evaluated with the Mini Nutritional Assessment short-form (MNA-SF), the Global Leadership Initiative on Malnutrition (GLIM), and the SARC-F questionnaire. Anthropometric and body composition measurements were also obtained. **Results**: Among 656 patients screened, 188 (28.6%) had dysphagia (median age 90 years; 89.9% females). Mild dysphagia was present in 34.0%, while 66.0% had moderate-to-severe dysphagia. At baseline, patients with moderate-to-severe dysphagia had significantly lower BMI (18.4 vs. 20.6 kg/m^2^, *p* = 0.014), smaller calf circumference (24.0 vs. 28.0 cm, *p* = 0.005), and higher SARC-F score (8 vs. 7, *p* = 0.028). Bioimpedance analysis showed lower fat mass (6.7 vs. 12.9 kg, *p* < 0.001) and fat mass% (14.7 vs. 25.4%, *p* < 0.001), and higher FFM% (85.3 vs. 74.6%, *p* < 0.001). At 6 months, BMI, calf circumference, handgrip strength, fat mass, and fat mass% resulted significantly lower in patients with moderate-to-severe dysphagia. A total of 23 participants (12.2%) died during follow-up, with a higher mortality rate in the moderate-to-severe group (HR 2.58, 95% CI 1.20–7.59, *p* = 0.044); aspiration pneumonia was the leading cause (21.7%). **Conclusions**: Dysphagia significantly affects nutritional status and survival in elderly residents of long-term care facilities. Early personalized nutritional intervention is pivotal to improve outcomes.

## 1. Introduction

Oropharyngeal dysphagia, defined as difficulty initiating or completing the act of swallowing due to dysfunctions in the oropharyngeal phase [1,2], is a common yet frequently underdiagnosed condition in older adults [2,3]. It poses a significant health concern, especially in long-term care facilities, where its reported prevalence ranges from 10% to 33% [4]. Swallowing efficiency tends to decline progressively with age due to a combination of neuromuscular, structural, and sensory changes affecting both the oropharyngeal and esophageal phases, often exacerbated by delayed diagnosis of esophageal motility disorders [5]. Specifically, swallowing is a tightly coordinated biomechanical process that integrates oral bolus preparation and propulsion, pharyngeal airway protection, and timely upper esophageal sphincter (UES) opening [6]. Age-related presbyphagia is characterized by reduced lingual pressure and endurance, delayed initiation of oropharyngeal swallowing, reduced laryngeal elevation, suboptimal epiglottic inversion and UES opening, slowed pharyngeal clearance, impaired UES relaxation, diminished laryngopharyngeal sensation, and often poor dentition with xerostomia [2]; these factors are frequently worsened by sarcopenia, multimorbidity, and polypharmacy [7,8]. As a result, older adults experience greater pharyngeal residue, increased risk of penetration–aspiration, and reduced airway protection; this deterioration can occur even in the absence of underlying diseases and contributes to a higher risk of malnutrition, aspiration, and poor quality of life [9,10,11].

Dysphagia is therefore associated with a cascade of adverse health outcomes, including malnutrition, dehydration, aspiration pneumonia, and increased mortality [12]. Its impact is particularly concerning in institutionalized populations, where it has been identified as a major risk factor for death [13]. Studies indicate that nearly half of dysphagic nursing home residents develop aspiration pneumonia within one year, with a mortality rate reaching up to 45% [14,15].

Among its complications, malnutrition is particularly common, as swallowing difficulties often result in insufficient oral intake, unintentional weight loss, sarcopenia, and functional decline [16,17]. The inability to consume a sufficient amount of nutrients further exacerbates frailty, contributing to poor clinical outcomes. In elderly individuals with dysphagia, the risk of malnutrition is significantly elevated. A study conducted in nursing homes found that 52% of individuals with dysphagia were malnourished compared to 17% of those without dysphagia [13]. Malnutrition negatively affects overall health and is associated with an increased risk of morbidity and mortality, further complicating disease management [18].

However, longitudinal data on the progression of nutritional decline in patients with dysphagia—and on the impact of dietary modifications and swallowing rehabilitation strategies on clinical outcomes—remain limited. Although various interventions, including texture-modified diets and swallowing therapy, are commonly employed, their long-term effectiveness remains insufficiently studied.

Moreover, dysphagia remains both common and underrecognized in long-term care settings. This is due to the frequent overlap of symptoms with frailty and dementia, inconsistent definitions and assessment tools across facilities, and irregular screening practices—further compounded by limited access to specialized care [19,20]. Recent evidence reports that over half of residents in aged care facilities experience dysphagia, with even higher prevalence observed in nursing homes when validated clinical assessments are used, leading to an increased risk of pneumonia, malnutrition, and mortality among older adults [21,22].

Given the significant clinical implications of dysphagia-related malnutrition, this study aims to evaluate the prevalence and progression of dysphagia among elderly residents in long-term care facilities in the Liguria region and to assess its impact on nutritional status and overall health outcomes.

## 2. Materials and Methods

This prospective, open-label, observational cohort study was conducted between January and June 2024 with the aim of evaluating the prevalence of dysphagia, as well as its nutritional and prognostic implications, among elderly residents in six long-term care facilities located in the Liguria region of Italy. 

The study was conducted in the following facilities: Joy S.r.l., Piccolo Cottolengo di Don Orione, Fondazione Chiossone, Istituto Martinez Casa per Anziani, Fondazione Casa di Riposo G.V.M. Macciò, and RSA La Doria.

We included patients aged ≥ 65 years with a documented clinical diagnosis of oropharyngeal dysphagia established by an otorhinolaryngologist, a phoniatrician, or a gastroenterologist with specific expertise in oropharyngeal swallowing disorders; eligible participants resided in one of the six participating long-term care facilities and were able to complete baseline bedside swallowing and nutritional assessments. We excluded patients in end-stage conditions with an expected survival < 3 months, those under exclusive palliative care, and patients with severe neurological impairment precluding a valid swallowing assessment, as well as participants with active solid malignancies or other obstructive conditions (e.g., advanced head-and-neck or upper aerodigestive tract masses) judged likely to markedly shorten survival or interfere with swallowing evaluation; we also excluded individuals with predominant esophageal dysphagia.

All residents aged ≥ 65 years in the participating facilities underwent screening with the 3 oz Water Swallow Test (WST). Participants with screen-positive or clinically documented oropharyngeal dysphagia were enrolled if baseline bedside swallowing and nutritional assessments could be completed. Dysphagia prevalence and severity (mild vs. moderate-to-severe) were evaluated at baseline using the WST. The patient was seated with the head in neutral; three 5 mL teaspoons of room-temperature water were administered with brief observation after each swallow, and—if passed—water was then offered directly from a cup; pass/fail and safety cues followed validated criteria (no pausing and no cough/choke or immediate wet/gurgly voice to pass; any such sign = fail/stop).

Severity categories were pre-specified as a bedside ordinal stratification, consistent with routine clinical practice and based on stepwise WST administration and safety cues: Grade 4 (severe dysphagia) = test stopped during the teaspoon phase because of severe cough and wet/gurgly voice; Grade 3 (moderate) = after cup drinking, concurrent wet/hoarse/gurgly voice and cough; Grade 2 (mild) = voice becomes wet/hoarse/gurgly without cough; Grade 1 (absent) = negative teaspoon and cup steps and a negative 50 mL continuous-drinking check [23,24,25].

At three months, participants underwent a standardized clinical reassessment.

Nutritional status was assessed using the Mini Nutritional Assessment—Short Form (MNA-SF) and, when applicable, the Global Leadership Initiative on Malnutrition (GLIM) criteria. Sarcopenia was screened using the Strength, Assistance in walking, Rise from a chair, Climb stairs, and Falls (SARC-F) questionnaire. Assessments were conducted by trained healthcare professionals, including dietitians and speech therapists specializing in dysphagia management.

Anthropometric and body composition parameters were collected at baseline and at 6 months. Body weight, height, and body mass index (BMI) were recorded alongside mid-upper arm and calf circumference measurements. Bioelectrical impedance analysis (BIA) was performed using the AKERN BIA 101 device (Akern srl, Florence, Italy), providing data on fat-free mass (FFM), fat mass (FM), total body water (TBW), body cell mass (BCM), phase angle (phA), skeletal muscle mass (SMM), and appendicular skeletal muscle mass (ASMM). These measurements were analyzed using dedicated software (Bodygram Plus^®^ v.1.0, Akern 2014, Firenze, Italy).

Clinical data, complications, and mortality during the follow-up period were systematically recorded.

During the 6-month follow-up period, dietary management followed routine clinical practices of the participating facilities; no dietary intervention was imposed by the study.

The study was conducted in accordance with the Declaration of Helsinki and was approved by the local Ethics Committee (N.CET-Liguria: SKK02/18, 25 June 2019). Written informed consent for data collection and analysis was obtained from all participants or their legal guardians. The study was reported in accordance with the STROBE guidelines (Table S1).

### Statistical Analysis

Continuous data were expressed as mean value ± standard deviation (SD) or as median and interquartile ranges (25th–75th percentile), while categorical variables were reported as absolute values and percentages. The normal distribution of the variables was tested using standard procedures such as Levene’s test and the Kolmogorov–Smirnov test. Comparisons of continuous variables were conducted using Student’s *t*-test, while changes over time were assessed through ANOVA. Discrete variables were compared using the χ^2^ test. Differences between groups were evaluated using unpaired *t*-tests, Wilcoxon, Mann–Whitney U (for variables not normally distributed), and Fisher’s exact tests (when comparing proportions), as appropriate. Relationships among variables were analyzed using the Spearman correlation coefficient.

Survival was calculated using the Kaplan–Meier method and was compared between groups using the log-rank test.

A two-tailed *p*-value < 0.05 was considered statistically significant. All statistical analyses were performed using SPSS v27.0 (Apache Software Foundation, Chicago, IL, USA), R (the R Project, R version 3.4.3; R Foundation for Statistical Computing, Vienna, Austria), and EZR (https://github.com/jinkim3/ezr (accessed on 11 May 2025).

## 3. Results

### 3.1. Baseline Characteristics

**Figure 1:**
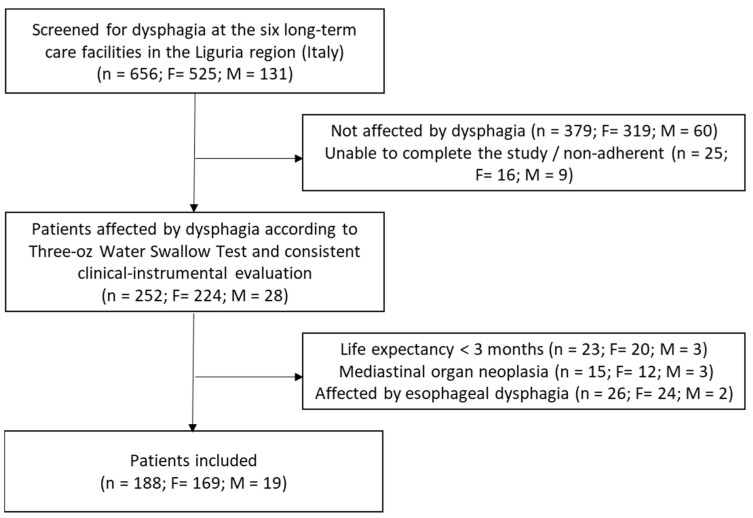
The flowchart summarizing the selection of the participants.

From an initial cohort of 656 patients screened (females: n = 525; males: n = 131), 188 (28.6%) were diagnosed with dysphagia and included in the analysis (Figure 1). The median age of the dysphagic population was 90 years, with a predominance of female patients (89.9%). The median MNA-SF score was 6 points, and GLIM was 1 point. Dysphagia was classified as mild in 34.0% of patients (n = 64) and as moderate-to-severe in 124 patients (66.0%). The baseline characteristics of the study population are shown in Table 1.

At baseline, patients with moderate-to-severe dysphagia showed significantly lower BMI (18.4, 15.7–21.6 vs. 20.6, 17.8–23.1 kg/m^2^, *p* = 0.014), reduced calf circumference (24.0, 21.5–27.0 vs. 28.0, 23.0–30.0 cm, *p* = 0.005), and higher SARC-F score (8, 8–8 vs. 7, 6–8 points, *p* = 0.028) compared to those with mild dysphagia. BIA revealed significantly lower FM in both absolute and relative terms among patients with moderate-to-severe dysphagia (FM: 6.7, 2.6–12.9 vs. 12.9, 8.1–18.7 kg, *p* < 0.001; FM%: 14.7, 7.1–26.8 vs. 25.4, 17.3–33.4%, *p* < 0.001). Patients with moderate-to-severe dysphagia also showed a higher FFM% (85.3, 73.2–92.9 vs. 74.6, 66.6–82.8%, *p* < 0.001) and TBW% (62.3, 53.9–69.2 vs. 57.2, 50.8–62.5%, *p* = 0.004). Additionally, ECW was significantly lower in the moderate-to-severe group (15.6, 14.1–17.9 vs. 17.2, 14.9–19.9 L, *p* = 0.024). No significant differences emerged between the two groups in terms of age, gender distribution, MNA-SF score, GLIM score, handgrip strength, and phase angle, as well as SMM and ASMM, which did not differ significantly between groups. Table 2 shows the overall baseline parameters according to dysphagia severity, with and without pair-matched comparisons (excluding deceased patients).

### 3.2. Anthropometrical and Body Composition Changes

At 6 months, BMI (17.0, 15.2–21.3 vs. 19.4, 17.1–23.1 kg/m^2^, *p* = 0.014), calf circumference (24.0, 21.0–27.0 vs. 26.5, 23.3–29.8 cm, *p* = 0.003), handgrip strength (3.4, IQR 1.0–6.8 vs. 5.2, 2.5–10.0 kg, *p* = 0.031), FM (7.5, 3.7–17.0 vs. 12.9, 8.4–19.5 kg, *p* = 0.024), and FM% (18.2, 8.8–29.9% vs. 25.5, 18.6–32.6%, *p* = 0.025) were significantly lower in patients with moderate-to-severe dysphagia compared to those with mild dysphagia. Conversely, FFM% was significantly higher in patients with moderate-to-severe dysphagia (*p* = 0.025). No significant differences between groups were observed in phA, SMM, ASMM, TBW, ECW, and ICW. Significant within-group changes from baseline to the 6-month follow-up were observed: in the mild dysphagia group, BMI reduced from 20.6, 17.8–23.1 to 19.4, 17.1–23.1 kg/m^2^ (*p* = 0.006), handgrip strength from 7.8, 1.0–11.3 to 5.2, 2.5–10.0 kg (*p* = 0.010), while ECW increased from 17.2, 14.9–19.9 to 18.0, 14.5–20.0 L (*p* = 0.034); in the moderate-to-severe dysphagia group, BMI dropped from 18.4, 15.7–21.6 to 17.0, 15.2–21.3 kg/m^2^ (*p* = 0.017), phA from 4.0, 3.3–5.4 to 3.7°, 3.2–4.1 (*p* = 0.029), FFM from 37.1, 34.4–42.1 to 36.6 kg, 33.1–38.9 (*p* = 0.005), and ASMM from 12.8, 11.0–15.6 to 12.1, 10.5–14.0 kg (*p* = 0.021). Additionally, a significant decrease in ICW% from 42.3%, 37.3–51.6 to 40.6%, 36.6–43.4 (*p* = 0.031), along with a corresponding increase in ECW% from 57.7%, 48.4–62.7 to 59.4%, 56.6–63.4 (*p* = 0.031), was observed (Table 3).

### 3.3. Mortality Outcomes

During the 6-month follow-up period, 23 patients (12.2%) died. The leading cause of death was aspiration pneumonia (n = 5, 21.7%), followed by cardiac disease (n = 4, 17.4%), senile dementia (n = 3, 13.0%), stroke (n = 2, 8.7%), and oncological conditions (n = 1, 4.3%). The remaining eight patients (34.8%) died from other causes. Table 4 reports the mortality and causes of death during the 6-month follow-up period among the study population.

**Figure 2:**
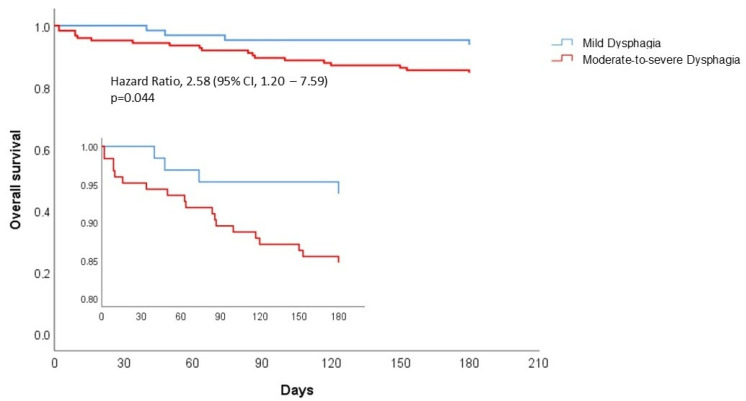
Kaplan–Meier curves for overall survival according to dysphagia severity.

Figure 2 illustrates the Kaplan–Meier survival curves comparing patients with mild dysphagia and moderate-to-severe dysphagia. Mortality was significantly higher in patients with moderate-to-severe dysphagia compared to those with mild dysphagia (HR 2.58, 95% CI: 1.20–7.59, *p* = 0.044).

## 4. Discussion

This prospective cohort study highlights the prevalence of dysphagia among elderly patients in six long-term care facilities in the Liguria region and its substantial impact on nutritional status, body composition, and clinical outcomes. In our cohort, the prevalence of dysphagia was 28.6%, which aligns with existing evidence. In a large observational study including 3451 European and Israeli nursing home residents aged over 65 years, the prevalence of dysphagia was 30.3% [26]. Similarly, a recent meta-analysis including 17 studies reported an overall prevalence of swallowing disorders of 33.2% among institutionalized elderly patients [27], further confirming the clinical relevance of dysphagia as a major health concern in this vulnerable population.

Our findings indicate that patients with moderate-to-severe dysphagia presented a markedly compromised nutritional status compared to those with mild dysphagia. Indeed, patients with moderate-to-severe dysphagia present a lower BMI than patients with mild dysphagia, reflecting a state of protein-energy malnutrition [28]. Although BMI alone may not fully address changes in muscle mass [29], it remains a widely used marker of protein stores [30]. The lower calf circumference observed in the moderate-to-severe group further supports the presence of sarcopenia, as this anthropometric marker is a well-established surrogate for peripheral muscle mass and function in older adults [31]. Moreover, decreased calf circumference has also been strongly associated with impaired physical performance, increased frailty, and higher mortality risk among older adults [32,33]. Additionally, the higher SARC-F score observed in patients with moderate-to-severe dysphagia emphasizes both muscle mass depletion and deterioration of nutritional status [34].

Body composition analysis through BIA showed that patients with moderate-to-severe dysphagia had significantly lower FM, FM%, and ECW volume, along with relatively higher FFM% and TBW% compared to those with mild dysphagia. While these latter findings might initially suggest a more favorable body composition, they are more likely to reflect a relative increase in lean compartments due to fat mass depletion rather than a true gain in muscle tissue or hydration. This interpretation is supported by the absence of significant differences in SMM and ASMM, muscle strength (handgrip), and phA between the groups. Furthermore, as Kyle et al. noted, bioelectrical impedance may yield inaccurate estimates of FFM and TBW in older or malnourished individuals, especially when fluid redistribution or altered body composition is present [35]. Similarly, another study emphasized that BIA data can be misleading under conditions of altered hydration, particularly when fat stores are depleted or fluid compartments are imbalanced [36]. Thus, the lower ECW volume in the moderate-to-severe dysphagia group, combined with preserved SMM, ASMM, handgrip strength, and PhA, suggests that muscle catabolism had not yet reached an advanced stage. This finding is consistent with data from Dey et al. [37], who reported that, in elderly individuals, early nutritional decline is often characterized by preferential FM loss prior to detectable reductions in lean mass. Therefore, our findings suggest that patients with moderate-to-severe dysphagia may be in an early phase of malnutrition, characterized primarily by adipose tissue depletion preceding the onset of muscle wasting.

Given these findings, it is not surprising that, over the 6-month follow-up period, we observed significant longitudinal changes in several nutritional and functional parameters, particularly among patients with moderate-to-severe dysphagia. In this group, significant reductions in BMI, phA, FFM, and ASMM, along with fluid compartment shifts—such as increased ECW% and decreased ICW%—suggest an involvement of lean tissue compartments and compromised cell membrane integrity. These changes likely reflect progressive nutritional deterioration with measurable effects on body composition. Moreover, the more pronounced reductions in BMI and calf circumference observed in patients with moderate-to-severe dysphagia, compared to those with mild dysphagia, further reinforce the link between dysphagia severity and worsening nutritional and functional outcomes. This pattern aligns with previous studies showing that dysphagia contributes to a gradual loss of muscle mass, altered hydration status, and overall functional impairment, ultimately exacerbating malnutrition in frail older adults [3,38,39,40].

Importantly, survival analysis confirmed that dysphagia severity is a strong and independent predictor of mortality. Indeed, we observed that patients with mild-to-moderate dysphagia exhibited significantly reduced survival compared to those with mild dysphagia. This finding is consistent with recent evidence showing that severe dysphagia is associated with a significantly higher risk of death than mild dysphagia [12]. Similarly, Shune et al. [41] found that patients with severe dysphagia experienced significantly reduced survival compared to those with mild dysphagia. These findings underscore the progressive nature of dysphagia-related complications, where advanced swallowing dysfunction may lead to repeated aspiration events, impaired nutritional intake, and systemic deconditioning, ultimately increasing the risk of death.

In light of these considerations, the present findings should be interpreted within the broader context of the dysphagia–sarcopenia–frailty triad, a geriatric syndrome that has gained increasing attention in recent years. Recent European and Asian cohort studies have demonstrated that nearly half of institutionalized older adults with dysphagia also meet criteria for sarcopenia—a condition now recognized as sarcopenic dysphagia [42]. Campo-Rivera et al. reported that this phenotype doubles the three-year mortality risk compared with dysphagia not related to sarcopenia [42]. Similarly, Celik et al. showed that dysphagia statistically mediates approximately 17% of the association between sarcopenia (as assessed by the SARC-F) and overall frailty in community-dwelling older adults [43]. These data support our observation that moderate-to-severe dysphagia is associated with early FM depletion and declining muscle performance even before ASMM reduction reaches diagnostic thresholds for sarcopenia. A noteworthy contribution from the recent literature is the concept of rehabilitation nutrition, which involves the integration of high-protein, high-energy dietary strategies (≥30 kcal/kg/day and ≥1.2 g protein kg/day) with intensive swallowing and resistance training [7,26,44]. Our 6-month trajectories—declining BMI, handgrip, and phA despite standard dietetic care—suggest that the nutritional regimen routinely provided in long-term care may be insufficient to prevent further decline, highlighting an area for improvement in current clinical practice.

In our study, the predominant cause of mortality among patients was aspiration pneumonia, a well-recognized yet often underdiagnosed complication in this population. This result is consistent with existing epidemiological data. For instance, Gonzalez-Fernandez et al. analyzed mortality records in the United States from 1999 to 2017 and identified aspiration pneumonia as the underlying cause of death in more than 330,000 cases, with patients aged 75 and older accounting for the vast majority of these deaths [45]. Likewise, Langmore et al. [46] demonstrated that dysphagia is an independent risk factor for aspiration pneumonia in the elderly. Despite being highly prevalent and clinically significant, aspiration pneumonia remains a frequently overlooked endpoint in dysphagic patients, especially in long-term care settings.

Our findings support the need for early identification of dysphagia in long-term care residents, with emphasis on targeted nutritional interventions. Individualized approaches may include the timely initiation of texture-modified diets and the administration of oral nutritional supplementation [47,48]. In patients with concurrent sarcopenia, strategies should also address protein intake and muscle preservation [49]. These interventions could be adapted to the degree of dysphagia, comorbidities, and functional status [50]. In line with prior studies, we observed that dysphagia was strongly associated with poorer nutritional status and increased mortality [12,13]. However, our data provide a more nuanced understanding of how dysphagia severity contributes to longitudinal deterioration in nutritional status, underscoring its central role in the nutritional decline observed in long-term care residents.

However, this study has several limitations. Dysphagia diagnosis was based solely on clinical assessment without instrumental confirmation, which may have led to misclassification in borderline cases. Furthermore, current evidence suggests that esophageal dysfunction, inflammatory phenotypes, and immuno-inflammatory pathways may also influence swallowing mechanics and nutrition-related outcomes [51,52,53,54,55]. Although body composition was thoroughly assessed, inflammatory markers were not evaluated, which could have provided further insights into the mechanisms underlying nutritional decline. Additionally, we did not collect standardized facility-level data on prescribed vs. achieved energy/protein intake or adherence to texture prescriptions. As recommended in recent evidence, integrated care bundles including nutritional monitoring (e.g., prescribed vs. achieved energy/protein targets), texture modification, swallowing rehabilitation, and strength training should be routinely implemented in long-term care [7,44,56,57,58]. The observational nature of the study precludes any causal inference, and the relatively short follow-up limits conclusions on long-term outcomes. In addition, given that most analyses were unadjusted, these findings should be considered associative rather than causal, and the presence of residual confounding cannot be excluded. Lastly, we were unable to collect comprehensive data on residents who screened negative for dysphagia due to feasibility constraints (including limited access to BIA devices and staff availability), which prevented the inclusion of a full control group for adjusted comparisons across the entire screened cohort.

Nevertheless, this study has several strengths. To our knowledge, it is one of the few prospective cohort studies specifically investigating the clinical, nutritional, and survival impact of dysphagia in a real-world long-term care setting. A multidimensional assessment approach was adopted, integrating bedside swallowing evaluation, standardized nutritional screening tools, functional status tests, and detailed body composition analysis. This multidimensional evaluation allowed for a nuanced characterization of the interplay between dysphagia severity, nutritional deterioration, and mortality risk. Moreover, the inclusion of a relatively large and homogeneous institutionalized elderly population provides relevant insights for clinical practice in geriatric care. When juxtaposed with multi-country surveillance programs such as SHELTER (prevalence 30.3%, one-year mortality OR 1.58) [26], our Ligurian prevalence (28.6%) and an HR of 2.58 fall within the expected range, reinforcing the external validity of our cohort. The concurrence of fat-mass loss, calf-circumference reduction, and elevated SARC-F in the moderate-to-severe subgroup highlights the progressive, nutrition-driven trajectory already recognized internationally.

## 5. Conclusions

In conclusion, our findings set against the current body of evidence confirm that dysphagia in long-term care is not merely an isolated swallowing disorder but the clinical nexus of malnutrition, sarcopenia, and geriatric frailty. The assessment of dysphagia severity could predict nutritional decline and mortality in long-term care, and residents with moderate-to-severe dysphagia therefore warrant early dietetic referral, texture and fluid adaptation, swallow therapy, and routine monitoring of nutritional parameters. Failure to recognize and treat this interconnected syndrome perpetuates a downward spiral of adipose- and lean-tissue loss, functional dependency, and mortality. Conversely, data accrued in the last years demonstrate that early dual-tool screening, rehabilitation nutrition, and structured exercise training—delivered by a coordinated multidisciplinary team—can arrest or even reverse this trajectory. Translating these findings into practice requires texture/fluid adaptation, explicit protein-energy targets, and multidisciplinary monitoring. Future research should evaluate the cost-effectiveness and long-term sustainability of such integrated programs.

## Figures and Tables

**Table 1:** Characteristics of the study population.

	Patients (n = 188)
Gender [F/M: n; %]	169 (89.9);19 (10.1)
Age [years] [IQR]	90 (85–94)
MNA-SF [points]	6 (5–8)
At risk of malnutrition (8–11 points)	39 (20.7)
Malnourished (0–7 points)	91 (48.4)
SARC-F [points]	8 (8–8)
GLIM [points]	1 (1–1)
Phenotypic criteria	
Low BMI (<22 kg/m^2^)	95 (55.2)
Non-volitional weight loss in past week	72 (37.8)
Reduced muscle mass	80 (62.0)
Etiological criteria	
Reduced food intake in past week	106 (56.4)
Disease burden/Inflammatory condition	21 (11.1)
Dysphagia level [n; %]	
Mild	64 (34.0)
Moderate-to-severe	124 (66.0)

Abbreviations: MNA-SF, Mini Nutritional Assessment—Short Form; SARC-F, Strength, Assistance with Walking, Rise from a Chair, Climb Stairs, and Falls (Sarcopenia Screening Tool); GLIM, Global Leadership Initiative on Malnutrition.

**Table 2:** Baseline clinical, anthropometric, and body composition parameters according to dysphagia severity, with and without pair-matched comparisons (excluding deceased patients).

Parameters	Mild (Pair-Matched)	Moderate-to-Severe (Pair-Matched)	*p*-Value (Pair-Matched)	Mild	Moderate-to-Severe	*p*-Value
Gender [F/M: n; %]	53 (88.3) 7 (11.7)	98 (93.3) 7 (6.7)	0.268	55 (85.9) 9 (14.1)	114 (91.9) 10 (8.1)	0.196
Age [years] [IQR]	91 (87–95)	89 (84–93)	**0.042**	90 (87–94)	89.5 (84–94)	0.343
Mini Nutritional Assessment [points]	6 (5–8)	6 (5–8)	0.550	6 (5–8)	6 (5–8)	0.648
SARC-F [points]	8 (7–8)	8 (8–8)	**0.018**	7 (6–8)	8 (8–8)	**0.028**
GLIM [points]	1 (1–1)	1 (1–1)	0.471	1 (1–1)	1 (1–1)	0.769
BMI [kg/m2]	20.5 (17.4–22.8)	18.8 (15.7–22.0)	0.054	20.6 (17.8–23.1)	18.4 (15.7–21.6)	**0.014**
Calf circumference [cm]	28.0 (22.5–29.0)	23.0 (21.5–27.5)	**0.019**	28.0 (23.0–30.0)	24.0 (21.5–27.0)	**0.005**
Hand grip strength [kg]	6.9 (1.0–11.0)	3.8 (1.6–6.0)	0.230	7.8 (1–11.3)	3 (1.6–5.6)	0.077
Phase angle [°]	3.7 (3.2–4.3)	4 (3.3–5.4)	0.073	3.7 (3.2–4.4)	4 (3.3–5.4)	0.103
Fat-Free Mass [kg]	38 (34.3–42.5)	37.1 (35.0–42.1)	0.612	38 (34.6–42.8)	37.1 (34.4–42.1)	0.469
Fat-Free Mass [%]	74.9 (66.7–83.0)	84.1 (72.2–92.6)	**0.003**	74.6 (66.6–82.8)	85.3 (73.2–92.9)	**<0.001**
Body cellular mass [kg]	14.8 (12.3–18.6)	15.6 (12.9–21)	0.222	14.8 (12.3–18.6)	15.5 (12.6–21)	0.336
Fat mass [kg]	12.4 (7.9–18.6)	7.0 (2.6–15.4)	**0.003**	12.9 (8.1–18.7)	6.7 (2.6–12.9)	**<0.001**
Fat mass [%]	25.0 (17.0–33.3)	16.0 (7.4–27.8)	**0.003**	25.4 (17.3–33.4)	14.7 (7.1–26.8)	**<0.001**
Total body water [L]	28.9 (25.9–32.2)	28 (25.5–31.2)	0.462	28.9 (26.1–32.4)	28 (25.4–31.2)	0.427
Total body water [%]	57.2 (51.1–62.4)	60.6 (53.6–68.5)	**0.018**	57.2 (50.8–62.5)	62.3 (53.9–69.2)	**0.004**
Extra-cellular water [L]	17.2 (14.9–19.7)	15.5 (14.1–17.7)	**0.018**	17.2 (14.9–19.9)	15.6 (14.1–17.9)	**0.024**
Extra-cellular water [%]	59.5 (54.9–64.0)	57.3 (48.8–62.3)	0.069	59.5 (54.4–64.4)	57.7 (48.4–62.7)	0.098
Intra-cellular water [%]	40.5 (36–45.1)	42.7 (37.7–51.2)	0.069	40.5 (35.7–45.7)	42.3 (37.3–51.6)	0.098
SMM [kg]	14 (12–19.3)	14.4 (12.5–17.5)	0.832	14 (12.1–19.3)	14.5 (12.4–17.6)	0.849
ASMM [kg]	12.7 (11–15)	12.9 (11.2–15.4)	0.931	12.7 (11–15.1)	12.8 (11–15.6)	0.765

Data are presented as absolute values and frequency, and as median and IQR. *p*-values refer to within-group paired comparisons. Bold values are statistically significant at *p* < 0.05. Abbreviations: BMI, Body Mass Index; SARC-F, Strength, Assistance with walking, Rise from a Chair, Climb Stairs, and Falls (Sarcopenia Screening Tool); GLIM, Global Leadership Initiative on Malnutrition; SMM, Skeletal Muscle Mass; ASMM, Appendicular Skeletal Muscle Mass.

**Table 3:** Anthropometric and body composition parameters according to dysphagia severity at 6 months.

Parameters	Mild	Moderate-to-Severe	*p*-Value *
BMI [kg/m^2^]	19.4 (17.1–23.1) †	17 (15.2–21.3) ‡	**0.014**
Calf circumference [cm]	26.5 (23.3–29.8)	24.0 (21.0–27.0)	**0.003**
Hand grip strength [kg]	5.2 (2.5–10.0) †	3.4 (1.0–6.8)	**0.031**
Phase angle [°]	3.7 (3.1–4.1)	3.7 (3.2–4.1) ‡	0.725
Fat-Free Mass [kg]	36.3 (34.8–42.3)	36.6 (33.1–38.9) ‡	0.772
Fat-Free Mass [%]	74.6 (67.4–81.5)	81.9 (70.1–91.2)	**0.025**
Body cellular mass [kg]	14 (11.7–17.8)	13.8 (12.1–17.3)	0.971
Fat mass [kg]	12.9 (8.4–19.5)	7.5 (3.7–17)	**0.024**
Fat mass [%]	25.5 (18.6–32.6)	18.2 (8.8–29.9)	**0.025**
Total body water [L]	28.1 (25.1–31.8)	27.4 (24.6–31.2)	0.469
Total body water [%]	55.9 (49.9–63.7)	59.9 (52.8–67.3)	0.068
Extra-cellular water [L]	18 (14.5–20.0) †	16.1 (14.7–18.0)	0.185
Extra-cellular water [%]	60 (56.8–64.7)	59.4 (56.6–63.4) ‡	0.713
Intra-cellular water [%]	40.1 (35.3–43.3)	40.6 (36.6–43.4) ‡	0.713
SMM [kg]	14.0 (11.5–17.0)	14.0 (11.7–16.9)	0.914
ASMM [kg]	12.2 (11.0–14.5)	12.1 (10.5–14.0) ‡	0.580

Data are presented as median and IQR. † statistically significant at *p* < 0.05 in patients with mild dysphagia between baseline and follow-up at the Wilcoxon test. ‡ statistically significant at *p* < 0.05 in patients with moderate-to-severe dysphagia between baseline and follow-up at the Wilcoxon test. * statistically significant at *p* < 0.05 between patients with mild dysphagia and moderate-to-severe dysphagia at Mann–Whitney. Bold values are statistically significant at *p* < 0.05. Abbreviations: BMI, Body Mass Index; FM, Fat Mass; FM%, Fat Mass Percentage; FFM%, Fat-Free Mass Percentage; TBW, Total Body Water; TBW%, Total Body Water Percentage; ECW, Extracellular Water; ECW%, Extracellular Water Percentage; ICW%, Intracellular Water Percentage; SMM, Skeletal Muscle Mass; ASMM, Appendicular Skeletal Muscle Mass; phA, Phase Angle.

**Table 4:** Mortality and causes of death during the 6-month follow-up period among the study population.

	Patients (n = 188)
Death during the 6-month follow-up period	23 (12.2)
Cause of death	
Senile dementia	3 (13.0)
Aspiration pneumonia	5 (21.7)
Cardiac disease	4 (17.4)
Oncological disease	1 (4.3)
Stroke	2 (8.7)
Other	8 (34.8)

Data are presented as absolute values and frequencies.

## Data Availability

The data presented in this study are available on request from the corresponding author due to ethical reasons.

## Supplementary Materials

The following supporting information can be downloaded at https://www.mdpi.com/article/10.3390/nu17203268/s1, Table S1: STROBE Statement.
